# Association of State COVID-19 Vaccination Prioritization With Vaccination Rates Among Incarcerated Persons

**DOI:** 10.1001/jamanetworkopen.2022.6960

**Published:** 2022-04-12

**Authors:** Breanne E. Biondi, Kathryn M. Leifheit, Carmen R. Mitchell, Alexandra Skinner, Lauren Brinkley-Rubinstein, Julia Raifman

**Affiliations:** 1 Department of Health Law, Policy and Management, Boston University School of Public Health, Boston, Massachusetts; 2 Fielding School of Public Health, Department of Health Policy and Management, University of California, Los Angeles; 3 Department of Health Management and System Sciences, University of Louisville School of Public Health and Information Sciences, Louisville, Kentucky; 4 Department of Social Medicine, University of North Carolina at Chapel Hill

## Abstract

This cross-sectional study examines the association between prioritization of COVID-19 vaccination for state prison systems and the rate of vaccination among incarcerated persons.

## Introduction

Risk of COVID-19 transmission is increased in prisons and surrounding communities. COVID-19 can spread rapidly in these facilities owing to crowding, inability to socially distance, poor ventilation, continuous admissions and releases, and daily work staff.^1^ High rates of chronic and immunocompromising conditions such as HIV among incarcerated persons are associated with greater risk of COVID-19.^2^

Owing to limited vaccine supply in December 2020 and early 2021, the Advisory Committee on Immunization Practices recommended that states allocate COVID-19 vaccines in phases and did not prioritize incarcerated persons for vaccination, nor did many states.^3^ In contrast, the National Academies of Sciences, Engineering, and Medicine recommended that incarcerated persons be vaccinated in the second phase.^4^ This study assessed COVID-19 vaccine rollout in state prison systems and the association between vaccination prioritization policies and the percentage of incarcerated persons vaccinated for COVID-19.

## Methods

This longitudinal cross-sectional study used state data on weekly COVID-19 vaccination counts among incarcerated persons and monthly prison population counts from the Marshall Project and Associated Press. Phases and dates of incarcerated persons' vaccination eligibility were obtained from the COVID-19 US State Policy database. Boston University’s institutional review board waived deemed the study non–human participants research and waived informed consent. We followed the STROBE reporting guideline.

The sample included states with complete data on incarcerated persons’ vaccination (eTable and eAppendix in the Supplement). The outcome was the cumulative percentage of fully vaccinated incarcerated persons per state. The exposure was the phase/date of incarcerated persons' vaccination eligibility. We estimated the exposure-outcome association using an event study analysis. For the exposure, we created binary indicators for weeks relative to vaccine prioritization, setting values to 0 for states that never prioritized incarcerated persons for vaccination. The event-study effect estimates represent absolute percentage-point differences in cumulative incarcerated persons’ vaccination rates in each week before and after prioritization vs the period immediately before.^5^ The analysis period was October 20, 2020, to June 20, 2021 (last week of available data); the unit of analysis was state-week. Models included fixed effects for state and week to control for time-invariant differences between treated and untreated states and national trends in incarcerated persons’ vaccination. A secondary analysis assessed June 2021 vaccination rates among incarcerated persons vs the general adult population by month of incarcerated persons’ vaccination prioritization.

## Results

**Figure 1.**
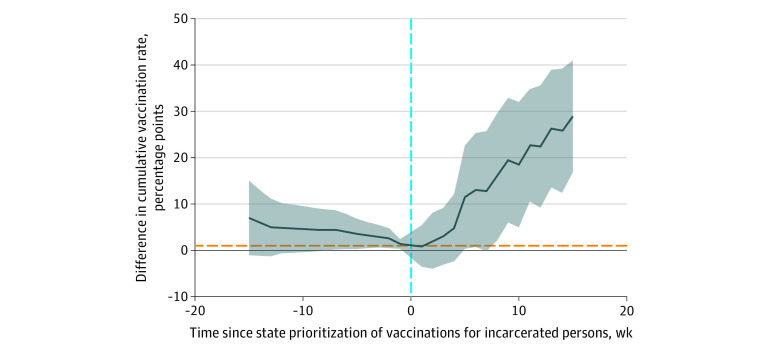
Associations Between State COVID-19 Vaccination Prioritization and Cumulative Vaccination Rate Among Incarcerated Persons in 36 States Event-study coefficients estimated the mean treatment effect in states that prioritized vaccination of incarcerated persons (ie, the absolute difference in the cumulative percentage vaccinated in states that prioritized vs did not prioritize vaccination for incarcerated persons). Fixed effects were used for state and week, and SEs were clustered at the state level. Shading indicates 95% CIs. The dashed horizontal line indicates no difference in vaccination rates among states with a policy prioritizing incarcerated persons to be vaccinated against COVID-19 compared with states that did not prioritize incarcerated persons to be vaccinated, and the dashed vertical line indicates the time point when a state created a policy prioritizing vaccination among incarcerated persons.

Of 36 analyzed states (mean, 690 343 incarcerated persons [range, 663 747-712 716]), 21 prioritized incarcerated persons for vaccination and 15 did not. Incarcerated persons became eligible for vaccination from December 12, 2020, to April 12, 2021. States with policies prioritizing incarcerated persons’ vaccination had significant increases in vaccination rates vs other states over time (Figure 1).

**Figure 2.**
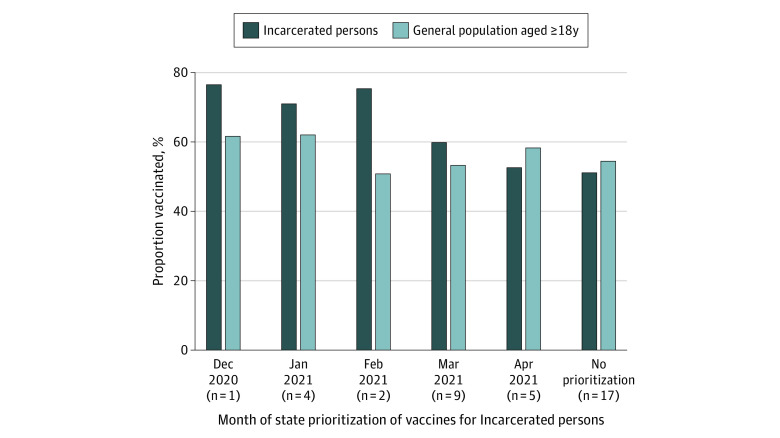
Vaccination Rates Among Incarcerated Persons and the General Population by Month of Vaccination Prioritization for Incarcerated Persons N values indicate the number of states in that month that prioritized vaccination of incarcerated persons.

Only 10 states vaccinated more than 70% of incarcerated persons (of these, 7 prioritized vaccinations for incarcerated persons), and only North Dakota fully vaccinated more than 80%. By June 2021, states prioritizing vaccinations for incarcerated persons earlier in 2021 had higher incarcerated persons’ vaccination rates vs states prioritizing incarcerated persons later or not at all and vs the general population (Figure 2). Even after prioritization, 42.0% of incarcerated persons remained unvaccinated in June 2021.

## Discussion

Low vaccination rates among incarcerated persons have implications for health equity. Racist policies in policing and drug criminalization sentencing created racial and ethnic disparities in incarceration and chronic disease, which are associated with increased risk of COVID-19 and complications among racial and ethnic minoritized individuals.^6^

Our data suggest that state prioritization of incarcerated persons was associated with increased vaccination rates in this population, although vaccination rates may vary owing to state vaccine rollout, availability, or incarcerated persons’ preference. Distrust of staff is common in prisons, and how to encourage vaccination among incarcerated persons should be considered.

Our analysis only included states that reported full doses of vaccination and thus may not be generalizable to the US prison population. Better data transparency, including full COVID-19 vaccinations and vaccinations by race and ethnicity, is needed to evaluate vaccinations in prisons.

## Notes

Supplement. **eTable.** States Used in Analysis and Prioritization Status **eAppendix.** Methods
eReferences
