# Supplementary material for: Association of State COVID-19 Vaccination Prioritization With Vaccination Rates Among Incarcerated Persons
Source: JAMA Netw Open. 2022 Apr 12;5(4):e226960. doi: 10.1001/jamanetworkopen.2022.6960 (PMC9006105; doi:10.1001/jamanetworkopen.2022.6960)
Supplement: Supplement. — eTable. States Used in Analysis and Prioritization Status eAppendix. Methods eReferences [file jamanetwopen-e226960-s001.pdf]

Supplementary Online Content

Biondi BE, Leifheit KM, Mitchell CR, Skinner A, Brinkley-Rubinstein L, Raifman J. Association of state COVID-19 vaccination prioritization with vaccination rates among incarcerated persons. *JAMA Netw Open*. 2022;5(4):e226960. doi:10.1001/jamanetworkopen.2022.6960

eTable. States Used in Analysis and Prioritization Status

eAppendix. Methods

eReferences

This supplementary material has been provided by the authors to give readers additional information about their work.

© 2022 Biondi BE et al. *JAMA Network Open*.

eTable. States Used in Analysis and Prioritization Status					
State	Used in Analysis	Prioritized Incarcerated Persons for Vaccination)	Vaccination Stage Prioritized	Month Prioritized	% of Incarcerated Persons Vaccinated (if used in analysis)
ALABAMA	no	yes	1B	Apr-21	
ALASKA	yes	yes	1B	Feb-21	67.7
ARIZONA	yes	yes	1C	Mar-21	72.5
ARKANSAS	no	yes	1C	Mar-21	
CALIFORNIA	yes	yes	1B	Mar-21	72.3
COLORADO	no	no	n/a	n/a	
CONNECTICUT	no	yes	1B	Apr-21	
DELAWARE	yes	yes	1C	Apr-21	47.6
FLORIDA	no	no	n/a	n/a	
GEORGIA	no	no	n/a	n/a	
HAWAII	yes	no	n/a	n/a	31.2
IDAHO	yes	yes	1C	Mar-21	48.6
ILLINOIS	yes	yes	1B	Jan-21	69.2
INDIANA	yes	no	n/a	n/a	54.6
IOWA	yes	yes	1B	Apr-21	66.4
KANSAS	no	yes	2	Jan-21	
KENTUCKY	yes	no	n/a	n/a	78.7
LOUISIANA	yes	no	n/a	n/a	66.8
MAINE	yes	no	n/a	n/a	73.5
MARYLAND	yes	yes	2	Apr-21	48.6
MASSACHUSETTS	yes	yes	1	Jan-21	69.8
MICHIGAN	yes	no	n/a	n/a	61.7
MINNESOTA	yes	yes	1B	Mar-21	68.1
MISSISSIPPI	yes	no	n/a	n/a	63.7
MISSOURI	yes	no	n/a	n/a	15.8
MONTANA	yes	yes	1C	Apr-21	27.4
NEBRASKA	no	yes	1C	Mar-21	
NEVADA	yes	no	n/a	n/a	8.0
NEW HAMPSHIRE	yes	no	n/a	n/a	77.0
NEW JERSEY	no	yes	1A	Dec-20	
NEW MEXICO	yes	yes	1B	Jan-21	74.8
NEW YORK	yes	yes	1B	Mar-21	51.6
NORTH CAROLINA	yes	yes	4	Mar-21	59.0
NORTH DAKOTA	yes	yes	1B	Feb-21	83.6

© 2022 Biondi BE et al. *JAMA Network Open*.

OHIO	no	no	n/a	n/a	
OKLAHOMA	yes	yes	2	Mar-21	54.6
OREGON	yes	yes	1A	Dec-20	76.7
PENNSYLVANIA	yes	yes	1B	Apr-21	73.7
RHODE ISLAND	yes	no	n/a	n/a	63.3
SOUTH CAROLINA	yes	yes	1B	Mar-21	55.1
SOUTH DAKOTA	no	no	n/a	n/a	
TENNESSEE	no	yes	3	Apr-21	
TEXAS	yes	no	n/a	n/a	43.4
UTAH	yes	no	n/a	n/a	6.7
VERMONT	yes	no	n/a	n/a	68.8
VIRGINIA	yes	yes	1B	Jan-21	70.8
WASHINGTON	no	yes	1B	Mar-21	
WEST VIRGINIA	yes	no	n/a	n/a	55.5
WISCONSIN	yes	yes	1B	Mar-21	61.2
WYOMING	no	yes	1C	Mar-21	

*Arizona's vaccination rates were not included in the Marshall Project dataset, and were obtained from https://corrections.az.gov/covid-19-management-updates

^ Kentucky's vaccination numbers from the Marshall Project only included partial vaccinations – these should be full vaccinations since Kentucky used the single-dose J&J vaccine (https://www.wdrb.com/news/coronavirus/kentuckys-prisons-to-allow-some-visitors-once-at-least-80-of-inmates-are-vaccinated/article_88978856-9bc3-11eb-8110-d32d761e8edf.html)

eAppendix. Methods

Data Sources

State data on weekly COVID-19 vaccination counts among ~~IP~~ incarcerated persons ~~-~~and monthly prison population counts were from The Marshall Project and Associated Press.[1] Monthly prison population counts were applied to each week.

State policies regarding vaccine allocation, from the COVID-19 US state policy database.[2]

COVID-19 Vaccinations in the United States, Centers for Disease Control and Prevention[3]

States that were excluded from the analysis did not report full vaccination rates (i.e. cumulative percent~~s~~ of incarcerated persons ~~IP~~ receiving two doses of Pfizer or Moderna vaccine or one dose of Johnson&Johnson), only partial (i.e. cumulative percent receiving at least one dose of vaccine). Nebraska did not report any vaccinations. For states that had missing cumulative vaccination data for a given week (N=57 of 1,294 state-weeks), we imputed the average of the previous and following week.

Analysis

The event study is used to analyze changes in outcomes in the pre- and post-periods by using binary indicators for weeks pre- and post- incarcerated persons ~~IP~~ vaccine eligibility date. Binary indicators for states that did not have a policy to prioritize incarcerated persons~~IP~~ for vaccination were all set to zero. The event study model allows us to analyze changes pre and post-policy change similar to a difference-in-differences analysis, except that the predicted outcome can vary across leads and lags. The estimated

effect (the percentage point difference in the cumulative percent of incarcerated persons~~IP~~ fully vaccinated) from the event study model are the effect of policies prioritizing incarcerated persons~~IP~~ to be vaccinated on the cumulative percent of incarcerated persons~~IP~~ vaccinated.

Analyses were performed using Stata/MP, v17.0 (StataCorp LLC, College Station, Texas).

Model Specification

(cumulative percent vaccinated)$_{st}$= (≤15 weeks prior)$_{st}$ + (14 weeks prior)$_{st}$ + (13 weeks prior)$_{st}$ + (12 weeks prior)$_{st}$ + (11 weeks prior)$_{st}$ + (10 weeks prior)$_{st}$ + (9 weeks prior)$_{st}$ + (8 weeks prior)$_{st}$ + (7 weeks prior)$_{st}$ + (6 weeks prior)$_{st}$ + (5 weeks prior)$_{st}$ + (4 weeks prior)$_{st}$ + (3 weeks prior)$_{st}$ + (2 weeks prior)$_{st}$ + (1 week prior)$_{st}$ + (1 week post)$_{st}$ + (2 weeks post)$_{st}$ + (3 weeks post)$_{st}$ + (4 weeks post)$_{st}$ + (5 weeks post)$_{st}$ + (6 weeks post)$_{st}$ + (7 weeks post)$_{st}$ + (8 weeks post)$_{st}$ + (9 weeks post)$_{st}$ + (10 weeks post)$_{st}$ + (11 weeks post)$_{st}$ + (12 weeks post)$_{st}$ + (13 weeks post)$_{st}$ + (14 weeks post)$_{st}$ + (≥15weeks post)$_{st}$ + (week)$_{t}$ + (state)$_{s}$ +ε_{st}

s=state
t=time (weeks)
(x weeks prior)$_{st}$= binary indicator of whether outcome was observed x weeks prior to the week incarcerated persons ~~IP~~ became prioritized for vaccination (among states that ever lifted; always 0 for states that never lifted)
(x weeks post)$_{st}$ = binary indicator of whether outcome was observed x weeks after the week incarcerated persons~~IP~~ became prioritized for vaccination (among states that ever lifted; always 0 for states that never lifted)
(week)$_{t}$ = fixed effects for calendar week
(state)$_{s}$ = fixed effects for state

References

1. *COVID Tracking in Prisons*. The Marshall Project; 2021. Accessed September 2, 2021. https://github.com/themarshallproject/COVID_prison_data

2. Raifman J, Nocka K, Jones D, et al. COVID-19 US state policy database. Published 2020. Accessed June 1, 2021. www.tinyurl.com/statepolicies

3. COVID-19 Vaccinations in the United States,Jurisdiction | Data | Centers for Disease Control and Prevention. Accessed September 2, 2021. https://data.cdc.gov/Vaccinations/COVID-19-Vaccinations-in-the-United-States-Jurisdi/unsk-b7fc

4. Goodman-Bacon A. *Difference-in-Differences with Variation in Treatment Timing*. National Bureau of Economic Research; 2018:w25018. doi:10.3386/w25018

© 2022 Biondi BE et al. *JAMA Network Open*.
